# Effect of variability of central venous pressure values to prevent atrial fibrillation after coronary bypass grafting

**DOI:** 10.22088/cjim.12.3.299

**Published:** 2021-04

**Authors:** Seyed Hossein Hamidi, Ghasem Faghanzadeh-ganji, Ali Baghaeian, Ali Bijani, Roghaieh Pourkia

**Affiliations:** 1 Clinical Research Development Unite of Rouhani Hospital, Babol University of Medical Sciences, Babol, Iran; 2 Student Committee Research, Department of Obstetrics and Gynecology, Babol University of Medical Sciences, Babol, Iran

**Keywords:** Central venous pressure, atrial fibrillation, coronary by pass grafting

## Abstract

**Background:**

Atrial fibrillation is an arrhythmia that results from abnormal depolarization of the atrium. Atrial fibrillation occurs in 5–40% of patients with cardiovascular bypass surgery, usually occurs on 2 to 4 days postoperatively. The aim of this study was Effect of variability of central venous pressure values to prevent atrial fibrillation after coronary bypass grafting.

**Methods:**

The present clinical trial study was performed on 150 patients undergoing cardiac surgery referred to Ayatollah Rohani Hospital of Babol. Patients were divided into 3 groups, with normal range pressure (8 to 12 mmHg), low pressure (less than 8), high pressure (greater than 12) based on central venous pressure measurements. Patients were evaluated every 4 hours to 72 hours for central venous pressure, AF incidence and urine output. Finally, the data are analyzed by spss statistical software.

**Results:**

In this study 79 (52.7%) patients were male and 71 (47.3%) were female. In examining changes in central venous pressure, the time effect also significantly increased central venous pressure. The results of independent t-test showed that the mean of central venous pressure changes in subjects with at day 16, second day at 16, 20, 24, third day at 4, 8, 12, 16, 20 and 24 hours Atrial fibrillation. Significantly more than those without atrialfibrillation (P<0.05).

**Conclusion:**

In the study, central venous pressure changes the effect of time significantly increases the central venous pressure. Individuals with atrial fibrillation also had significantly greater central venous pressure changes than those without atrial fibrillation.

Coronary artery bypass graft (CABG) surgery depends on the severity of the symptoms, coronary anatomy, and left ventricular function. Candidate patients have problematic or debilitating symptoms that, with medical treatment, are not completely controlled, or they cannot tolerate medical treatment and want to live a more active life, or stenosis in several branches of the coronary artery. They have severe. Atrial fibrillation is the most common postoperative arrhythmia that results from abnormal depolarization of the atrium, leading to a lack of atrial contraction (1-3). This complication is a potential for prolonging the hospital stay in the hospital and also for neurological and renal complications. The prevalence of atrial fibrillation varies from 5-40% after coronary artery bypass graft alone to 40% after valvular surgery and 50% after concurrent coronary and valve bypass surgery (4).

Atrial fibrillation has been reported between 12-7% in patients undergoing non-cardiac surgery (5). The time of onset of this complication is between 2-4 days after surgery and the maximum onset is the second day after surgery. In 90% of patients who suffer from this complication, this condition continues until the fourth day and in 94% until the end of the sixth day (6). Postoperative atrial fibrillation is an independent factor for mortality (7). For these reasons, finding a way to prevent the occurrence of this complication can be effective in reducing the length of hospital stay and the other complications, and also reduce costs. Several factors are effective in the development of atrial fibrillation after surgery.

 Atrial fibrillation in the postoperative period of CABG, due to the increased risk of congestive heart failure and embolic events, especially stroke, in the long run leads to worsening of patients' hemodynamic conditions. Strokes are so serious complications that are seen in 2% of surgical patients. In addition, atrial fibrillation is associated with increased hospital mortality and worsening long-term prognosis (3). One of the causes of atrial fibrillation is an increase in atrial size following volume administration and an increase in central venous pressure (8).

An important indicator of right ventricular filling pressure is the right ventricular filling pressure, ie the amount of blood pumped with each heartbeat (9). It is also an accurate indicator of the heart's ability to pump blood to maintain normal blood pressure and tissue perfusion. Finally, CVP is considered the correct indicator of right ventricular diastolic end volume (10). CVP is measured using a Central Venous Catheter. One end of the CVC is connected to an electronic manometer or transducer, computer, or monitor. Ultrasound may be used as a guide for CVC entry (11).

CVP monitoring is more accurate than measuring blood pressure, because changes in circulating volume are reflected in the CVP as soon as the blood volume decreases, and the CVP changes rapidly. The first stage of shock is followed by blood loss, while the blood volume decreases, the compensatory mechanisms act and the blood pressure remains normal, and only the CVP shows this decrease, while blood pressure is normal. CVP increases when there is an increase in the volume of the circulatory system or heart failure, and CVP decreases as result bleeding or fluid shifts inside the body compartments or volume loss (such as shock or as diabetes insipidus) (9, 12). The patient usually develops respiratory symptoms when the CVP is increasing. Conversely, when CVP is decreasing, urine volume may decrease and the patient may complain of severe thirst (12). Nowadays, cardiovascular diseases, including coronary heart disease, are one of the leading causes of death in humans worldwide (13). According to the World Health Organization, it will be the leading cause of death worldwide by 2020 (14). In the Eastern Mediterranean and the Middle East, including our country, cardiovascular disease is a major health and social problem, the rate of which is increasing rapidly.

In sporadic studies in Iran, 25 to 45% of the relative deaths were due to cardiovascular disease (15). Coronary heart disease, as one of the most common cardiovascular diseases, causes various complications such as myocardial infarction, angina pectoris and heart failure, each of which is a health problem (16). Atherosclerosis mainly causes angina pectoris and myocardial infarction, which is one of the most common diagnoses in hospitalized patients in industrialized countries (17). It causes more death, disability and cost than other diseases, so that ischemic heart disease is the most common cause of death in both men and women if all age groups are considered (17).

Coronary artery bypass graft surgery is one of the most valuable treatments that, if performed in a timely manner, can play a key role in reducing mortality and complications from these diseases. In this operation, which may be open or closed heart surgery, a bypass is created between the blocked arteries to supply blood to the heart muscle. Approximately 598,000 coronary artery bypass graft surgeries are performed annually in the United States alone, and although much progress has been made in drug therapy and catheterization procedures, surgical interventions are still the bases for the treatment of these diseases (18).

A 2017 study by Costa MACD et al. in Brazil found that keeping the central venous pressure lower (group 15 with central venous pressure) in the first 72 hours after surgery was relatively less risky. It is from POST CABG AF and can even prevent arrhythmias (1). A 2012 study by Wen-Hwa et al. In China. They compared two groups of people with POST CABG AF and those without POST CABG AF who underwent heart surgery and concluded that left atrial dilatation was an independent factor in the development of POST CABG AF and nosocomial mortality (p = In a 2004 study in Brazil by Silva et al., They reported that the incidence of POST CABG AF was high, significantly increasing morbidity and mortality and increasing the length of hospital stay. It was also said that among the independent factors, increasing fluid intake has a more prominent role (19). In a study conducted by Judson et al. In 2015 in the United States, which randomly selected 2390 patients at high risk of surgery or at the same time, they evaluated valvular problems and underwent coronary artery bypass grafting and concluded that central venous pressure in the first six hours after surgery, it was associated with mortality for the first 30 days as well as length of hospital stay (p = 0.001) (20). Due to the limited studies conducted in this field and the fact that such a study has not been done in Iran, so we decided to measure the incidence of AF by controlling the central venous pressure in the normal range to be able to measure the results of this study to prevent or minimize occurrence of POST CABG AF.

## Methods

This study was performed on patients who referred to Rouhani Hospital for open heart surgery between 2018 and 2019, after obtaining permission from the ethics committee of Babol University of Medical Sciences with the ethics code: IR.MUBABOL.HRI.REC.1398.009. The sample size was assumed to be 40% in AF and with 95% confidence level and 8% accuracy, with the following formula, the sample size was calculated as 145 patients, which we collected a total of 150 patients.

$n=\frac{{\left(Z_{1-\propto /2}+Z_{1-\beta }\right)}^{2}(S_{1}^{2}+S_{2}^{2})}{{\left(\mu_{1}-\mu_{2}\right)}^{2}}$

Inclusion criteria for patients who are candidates for open heart surgery and exclusion criteria’s, were patients with large atrium (either right or left), presence of moderate to high heart failure at the same time (mean ejection fraction less than 35%), the presence of AF before Operation, previous renal, hepatic, pulmonary disease, moderate to upper heart valve disorders, and peripheral vascular disease were considered. Then patients whom underwent CABG surgery and were transferred to the intensive care unit Based on central venous pressure measurements, patients were divided into two groups: the group with increased or decreased central venous pressure during hospitalization. With treatment, we normalized the central venous pressure in both groups, so that in the low-pressure group, giving fluids and, if necessary, vasopressor, and in the high-pressure group, limiting fluids and giving IV Furosemide. Then we examined the patients every 4 hours to 72 hours (3 days and nights), in terms of central venous pressure digitally with Saadat computer device and also continuous cardiac monitoring with 12-lead electrocardiogram device in terms of AF incidence and urine output control. The data and obtained from the two groups were analyzed by SPSS statistical software and when comparing the data, if the data distribution is normal, t-test and Chi-square, and if the data distribution is not normal, from Non-parametric tests were used. P value less than 0.05 was considered significant. Normal (8 to 12 mm Hg), low pressure group (less than 8), high pressure group (more than 12).

## Results

In this study, 150 patients with cardiovascular bypass surgery were studied, of which 79 (52.7%) were male and 71 (47.3%) were female. 16 patients (10.7%) had atrial fibrillation and 134 patients (89.3%) did not develop this complication. The minimum age of patients was 41 years and the maximum age was 85 years and the mean age of patients was 65.30 years with a standard deviation of 8.76 years Table (1-4).

**Table 1:** Demographic characteristics of research subjects

variable		Frequency	Percentage
Sex	male	79	52.7
	female	71	47.3
Atrial fibrillation	yes	16	10.7
	no	134	89.3
Age ( SD ± Mean)		8.79± 65.30	

In men, 5 patients (6.3%) and in women, 11 patients (15.5%) developed atrial fibrillation, which did not show a statistically significant difference (P=0.07). In the study of mean age between people with atrial fibrillation and those who did not, it was found that people with atrial fibrillation had a mean age of 65.50 7 7.27 and those who did not have atrial fibrillation had a mean age of 65.16 94 8.94 This difference was not statistically significant (P=0.56) (Table 2-4).

**Table 2:** Relationship between age and sex variables with atrial fibrillation

**AF**		**Yes** **Frequency (%)**	**No** **Frequency (%)**	**Meaningfulness**
**Variable**				
sex	male	5 (6.3)	74 (93.7)	0.07
	female	11 (15.5)	60 (84.5)	
SD)±Age)		7.27±65.50	8.94±65.16	0.56

**Figure 1:**
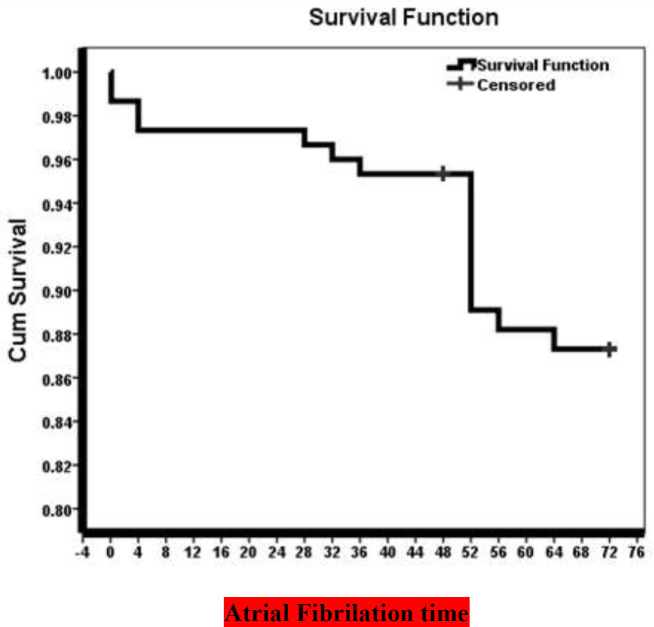
Chart of atrial fibrillation time in patients by Kaplan Meyer method

In estimating the mean time of atrial fibrillation in patients using Kaplan Meyer method, the mean time was equal to 77.0 hours with a standard deviation of 5.72 hours which can be seen in Table 3 and Figure 1.

**Table 3:** Mean and 95% confidence interval of atrial fibrillation

Mean Time			
Estimation	SD	95% Confidence	
		Lower limit	Upper limit
77.0	5.72	36.42	58.86

In the study of changes in central venous pressure, the results of analysis of variance with repeated data showed that the effect of time significantly increases the central venous pressure (P<0.001, df=18, F=36.29). Also, patients with atrial fibrillation had significantly more changes in central venous pressure than those without atrial fibrillation (P<0.001, df = 1, F=15.90). Finally, the interaction between time and atrial fibrillation was also reported to be significant (P<0.001, df=18, F=3.41).

The results of independent t-test showed that at the time of the first day at 16:00, the second day at 16, 20, 24, the third day at 4, 8, 12, 16, 20 and 24 the average changes in central venous pressure in people with Atrial fibrillation is significantly higher than people who did not have atrial fibrillation (P<0.05) (Table 3).

**Table 4:** Mean changes in central venous pressure in individuals with and without atrial fibrillation

Central vein Pressure	Atrial Fibrillation		Meaningfulness
	yes Mean±SD	No Mean±SD	
First day Hour 0	92/1 ± 68/9	91/1±17/10	33/0
First day Hour 4	22/2±43/10	25/2±52/10	88/0
First day Hour 8	04/2±06/11	34/2±98/10	90/0
First day Hour 12	67/1±43/12	17/2±45/11	08/0
First day Hour 16	52/2±12/13	0/2±63/11	007/0
First day Hour 20	06/2±0/13	26/2±27/12	22/0
First day Hour 24	82/1±56/13	28/2±70/12	15/0
Second day Hour 4 4	27/2±43/14	27/2±53/13	13/0
Second day Hour 8	28/2±50/14	62/2±55/13	16/0
Second day Hour 12	06/2±0/15	45/2±90/13	08/0
Second day Hour 16	90/2±25/15	54/2±88/13	04/0
Second day Hour 20	97/2±93/15	41/2±76/13	001/0
Second day Hour r 24	72/2±31/15	38/2±43/13	004/0
Third day Hour 4 4	37/2±81/14	0/2±15/13	003/0
Third day Hour 8	44/2±31/15	75/1±12/13	003/0
Third day Hour 12	98/2±31/15	84/1±95/12	007/0
Third day Hour 16	96/2±0/15	65/1±91/12	01/0
Third day Hour 20	07/3±37/15	72/1±85/12	005/0
Third day Hour 24	46/2±25/15	72/1±64/12	001/0

**Figure 2:**
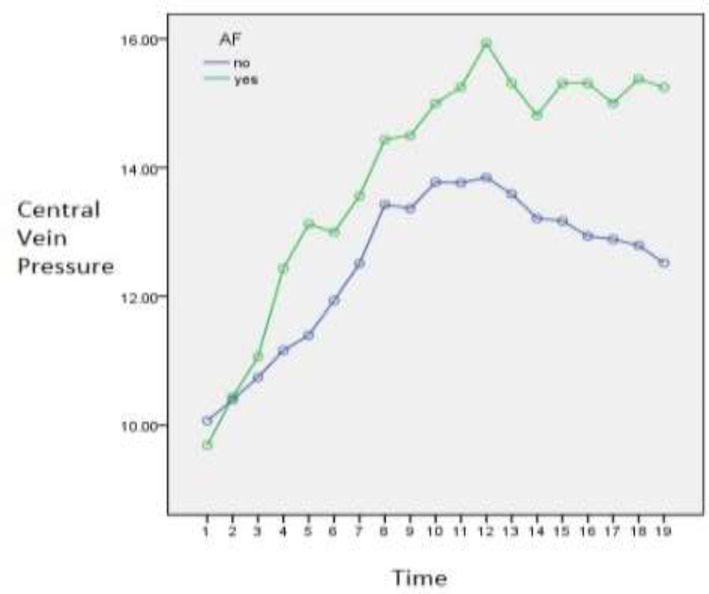
Diagram of changes in central venous pressure in individuals with and without atrial fibrillation

## Discussion

In a study conducted by Costa MACD et al. In 2017 in Brazil entitled "Comparison of the effect of two strategies of central venous pressure control on the incidence of POST CABG AF" on 140 patients in two groups of 70, one with central venous pressure 15 and Another was performed with a central venous pressure of 20, concluding that keeping the central venous pressure lower (group with a central venous pressure of 15) in the first 72 hours after surgery was relatively less risky than POST CABG AF, ​​and even Can prevent arrhythmia, which in our study also showed that people with atrial fibrillation had significantly more changes in central venous pressure than people without atrial fibrillation. The study also showed that the average age of people was between 60-63 years (1). 

In our study, the mean age of individuals in the two groups with and without fibrillation was 65 years, with no statistically significant difference between the two groups. While age over 65 is one of the most important risk factors for atrial fibrillation after CABG, age is associated with myocardial structural changes due to degeneration processes (fibrosis and dilation), which lead to abnormal resistance and conduction and atrial fibrillation is possible.

A 2012 study by Wen-Hwa et al. In China entitled "Left Atrial Dilatation, a Criteria for POST CABG AF and Hospital Mortality after Cardiovascular Reconstruction Surgery" was performed on 197 patients without known valvular problems. They compared two groups of people with POST CABG AF and those without POST CABG AF who underwent heart surgery and concluded that left atrial dilatation was an independent factor in the development of POST CABG AF and nosocomial mortality (p = Also in this study, the results showed that among the factors associated with atrial fibrillation after CABG age, male gender (21). In our study, although the number of men who underwent CABG surgery was higher than women, the rate of atrial fibrillation was higher in women than men, but there was no statistical difference between them. This could indicate that gender could not be a significant risk factor for postoperative AF.

In a 2015 study by Judson et al. In the United States, a statistical analysis of the study entitled "Can central venous pressure after vascular bypass surgery predict mortality and postoperative renal failure?" Randomly evaluated 2390 patients at high risk of surgery or at the same time with valvular problems and underwent coronary artery bypass grafting and concluded that central venous pressure in the first six hours after surgery with a mortality of 30 days First and also related to the length of hospital stay (p = 0.001) (20).

Nezam Ahmadi et al. In 2012, in a study aimed at investigating hemodynamic changes after coronary artery bypass grafting and related factors in patients undergoing open heart surgery admitted to the intensive care unit of cardiac surgery, showed results that showed the amount of pressure Central vein above normal within 24 hours after patient admission, decrease and decrease in central venous pressure below normal at 24 hours after patient admission, indicating improvement in central venous pressure. The highest frequency of central venous pressure above normal was related to the time 2 hours after admission to the ward (8.28%) and the highest frequency in patients with lower than normal central venous pressure was related to the time 12 hours after admission to the ward. (6.26%) Also, the highest frequency of normal central venous pressure is related to the time 4 hours after the patient enters the ward (22). While the results of our study, changes in central venous pressure, based on the results of analysis of variance, showed that the effect of time significantly increases the central venous pressure.

Alavi et al. Conducted a study in 2016 with the aim of comparing the index of pulse pressure fluctuations and central venous pressure in assessing fluid volume and its optimal optimization in patients after heart surgery. Their results showed that the rate of changes in central venous pressure after from receiving the volume of fluids has reached a considerable level. However, this increase was not in line with the increase in cardiac index and other cardiac function indices, and it can be concluded that any increase in central venous pressure is associated with an increase in these indices, which was not the case, and it can be concluded that any increase Significantly (statistically) in central venous pressure cannot be clinically effective in improving the prognosis of patients (23). 

But in our study, the results showed that people who had atrial fibrillation had significantly more changes in central venous pressure than people who did not have atrial fibrillation. Evaluating fluids and determining intravascular volume clinically in critically ill patients undergoing major surgery such as heart surgery is a major challenge. The purpose of assessing intravascular volume status in patients with hemodynamic instability is primarily to determine whether they benefit from fluid administration. Among the limitations of this study, blinding was not performed in the CVP evaluation of this study. Thus, physicians' responses to CVP values ​​may lead to underestimating the relationship between CVP and outcome.

In conclusion CABG is associated with postoperative complications and risks, although its advantages far outweigh its disadvantages, so it is important to know enough about these complications and related factors to get the best results to increase patient survival. Found. The results of the Current study showed that in the study of changes in central venous pressure, the effect of time significantly increases the central venous pressure, so that on the third day the mean changes in central venous pressure in people with atrial fibrillation are significantly higher than those who They did not develop atrial fibrillation. Also, people with atrial fibrillation had significantly more changes in central venous pressure than people who did not.
